# Clinical Characteristics and Outcomes of Patients Hospitalized with Epidermolysis Bullosa: A Retrospective Population-Based Observational Study in Spain (2016–2021)

**DOI:** 10.3390/biomedicines11092584

**Published:** 2023-09-20

**Authors:** Natividad Cuadrado-Corrales, Ana Lopez-de-Andres, Valentín Hernández-Barrera, David Carabantes-Alarcon, Jose J. Zamorano-Leon, Ricardo Omaña-Palanco, Jose L. Del-Barrio, Javier De-Miguel-Díez, Rodrigo Jimenez-Garcia, Juan J. Montoya

**Affiliations:** 1 Department of Public Health & Maternal and Child Health, Faculty of Medicine, Universidad Complutense de Madrid, 28040 Madrid, Spain; mariancu@ucm.es (N.C.-C.); dcaraban@ucm.es (D.C.-A.); josejzam@ucm.es (J.J.Z.-L.); romana@ucm.es (R.O.-P.); rodrijim@ucm.es (R.J.-G.); 2 Preventive Medicine and Public Health Teaching and Research Unit, Health Sciences Faculty, Rey Juan Carlos University, 28922 Madrid, Spain; valentin.hernandez@urjc.es (V.H.-B.); jose.delbarrio@urjc.es (J.L.D.-B.); 3 Respiratory Care Department, Instituto de Investigación Sanitaria Gregorio Marañón (IiSGM), Hospital General Universitario Gregorio Marañón, Universidad Complutense de Madrid, 28007 Madrid, Spain; javier.miguel@salud.madrid.org; 4 Faculty of Medicine, School of Sport Medicine, Universidad Complutense de Madrid, 28040 Madrid, Spain; jjmontoy@ucm.es

**Keywords:** epidermolysis bullosa, comorbidity, in-hospital mortality, length of hospital stay, cost of illness

## Abstract

(1) Background: Epidermolysis bullosa (EB) comprises a group of rare skin diseases. We assessed diagnostic procedures, comorbidity profiles, length of hospital stay (LOHS), costs, and in-hospital mortality (IHM) associated with EB. (2) Methods: A retrospective, population-based observational study was performed using the Spanish National Hospital Discharge Database. Hospitalized patients with EB in Spain were identified for the period 2016 to 2021. (3) Results: A total of 677 hospitalizations were identified among 342 patients with a diagnosis of EB. Fifty percent of patients had at least one readmission during the 6-year follow-up. Notably, rehospitalizations were more common among patients aged 2–17 years. The most prevalent comorbidity was digestive disorders, which were associated with the frequency of esophageal dilatation procedures and percutaneous endoscopic gastrostomy. The longest LOHS was recorded for the 0- to 1-year age group. IHM increased with age, and the difference was statistically significant. The prevalence of malignant neoplasm was 36.88%, with cutaneous squamous cell carcinoma being the most frequent. The overall cost per hospitalized patient was estimated to be EUR 10,895.22 (SD EUR 13,394.81), with significant variations between age groups. (4) Conclusions: Readmissions are very frequent among people with EB. We observed a higher LOHS in the 0- to 1-year age group, while the highest rates of IHM were observed in patients older than 50 years. There was a substantial prevalence of comorbidities, namely, digestive disorders, infectious diseases, and especially cancer.

## 1. Introduction

Rare diseases affect fewer than five out of every 10,000 individuals. Today, over 8000 rare diseases are known to exist. In the European Union alone, these diseases affect more than 30 million people. Health organizations throughout the world consider rare diseases to be a priority [1,2,3].

Epidermolysis bullosa (EB) comprises a heterogeneous group of monogenic skin diseases [4]. This cluster of rare diseases is characterized by blistering and scarring of skin and mucosal membranes caused by minimal trauma [5].

Over 1000 mutations have been identified in at least 16 genes expressed at the protein level at the dermo-epidermal junction. Depending on the degree of blister formation, the clinical categories defined for EB are dystrophic epidermolysis bullosa (DEB), epidermolysis bullosa simplex (EBS), and junctional epidermolysis bullosa (JEB) [6].

There are notable disparities in the estimated prevalence and incidence rates of EB among various populations. In the United States, the prevalence of EB is 11.1 per 1 million live births, with an incidence rate of 19.57 per 1 million live births [7]. Prevalence and incidence values are 15.4 and 20.1 per 1 million live births, respectively, in Italy [8,9] and 5.6–7.8 and 3.8 per 1 million live births in Japan [10,11]. Unfortunately, the epidemiological indices of EB in Spain are not well known, although data on the prevalence of DEB suggest a rate of six per 1 million live births [12].

The severity of clinical symptoms across different EB subtypes significantly impacts life expectancy. EB, and specifically DEB, can limit daily activities and shorten life expectancy [13]. Clinical manifestations, morbidity, and mortality in affected patients are consequences of cutaneous and mucosal involvement, the main comorbidities being cancer, gastrointestinal diseases, genitourinary diseases, kidney failure, cardiomyopathy, respiratory insufficiency, anemia due to chronic malnutrition, and growth failure [14,15]. The demanding and continuous care required for these conditions can result in substantial clinical and financial burdens for both patients and healthcare systems [16].

Given the rarity of EB, conducting studies with sufficient sample sizes is challenging, thus limiting our understanding of epidemiological patterns and hospital outcomes across all age groups. The complex diagnosis and diverse clinical manifestations of these conditions highlight the need for further research in epidemiology, diagnostic profiles, and hospital procedures [17]. Accurate epidemiological data are crucial if we are to understand this disease and can help us to select specialized medical care, assess the cost burden, and optimize resource allocation and funding. While hospital admissions for EB provide valuable insights into trends and outcome predictions, the currently available data in this area are limited and inconclusive [18].

The Spanish healthcare system, primarily funded through the national social security system, offers comprehensive coverage to residents and citizens. This coverage includes free-of-charge access to a wide range of medical services, hospital care, surgical treatments, and medications including special dressing agents such as silicon/polyurethane foam. The system is known for its inclusivity and equity, aiming to guarantee that healthcare services are accessible to all, regardless of their health status and socioeconomic status.

The objectives of this study were as follows: (1) To determine the number of hospitalizations with EB in Spain over a 6-year period and identify the total number of affected patients to assess changes over time. (2) To evaluate the characteristics of patients with EB, including demographics and clinical features, and analyze any changes over time. (3) To assess the length of hospital stay (LOHS) among EB patients and identify significant trends and variations. (4) To investigate in-hospital mortality (IHM) rates among EB patients and determine factors associated with increased risk. (5) To determine the prevalence of malignant neoplasms as comorbidities in patients with EB. (6) To identify and examine the most frequent procedures conducted during hospitalization for EB. (7) To analyze the costs associated with hospitalizations for EB and identify any notable patterns or changes.

## 2. Materials and Methods

The minimum basic data set (MBDS) is a system used by the Spanish National Health Service to collect and store patient information on hospital discharges. It gathers patient clinical, administrative, and financial data, such as diagnoses (up to 20), diagnostic, and therapeutic procedures (up to 20), LOHS, and billing. The International Classification of Diseases, Tenth Revision, Clinical Modification (ICD-10-CM) is used for coding [19]. The database incorporates all public and private hospital data and covers >95% of hospital discharges [20].

These anonymized data can be used for various purposes, including epidemiological research, health service planning, and cost analysis [21].

We designed a retrospective, descriptive, epidemiological study using data from the MBDS for the years 2016 to 2021. We selected all hospitalizations with an ICD-10-CM for EB (Q81.0, Q81.1, Q81.2, Q81.8, and Q81.9) in any diagnostic field. The MBDS includes a unique patient identifier, which was used to identify readmission over the 6-year period. The number of admissions was categorized as one, two, three, or more. Study covariates included age, sex, comorbidities, and procedures conducted during admission.

The modified Charlson comorbidity index (CCI) was calculated to assess the burden of comorbidities of each patient using the ICD-10-CM codes [22]. We have identified the presence of CCI conditions for each admission based on the information recorded in the patients’ discharge reports. The MBDS includes all diagnoses that were present at the time of admission or developed during the hospitalization. If the same individual was admitted multiple times, we have considered that they have all the comorbidities that were recorded in any of their admissions; therefore, the time period considered for calculating the CCI has been the 6 years of the study.

We categorized the patients into three groups: 0 (no disease), 1 (one disease), and 2 (two or more diseases).

In addition, we analyzed other previously described chronic and potentially severe comorbidities among EB patients, such as digestive diseases, respiratory diseases, infectious diseases, musculoskeletal diseases, and accidental poisoning [14,15]. Moreover, we specifically identified types of malignant neoplasm. Table S1 displays the specific comorbidities and types of malignant neoplasm identified in this study, accompanied by their corresponding ICD-10-CM codes.

Procedures included esophageal dilation, percutaneous endoscopic gastrostomy surgery, excision or extraction of skin, replacement of skin with nonautologous/autologous tissue substitute, extraction and delivery of products of conception, transfusion, and skin care treatments and procedures. Table S2 presents the ICD-10-CM codes for the seven procedures performed during hospitalization in this study.

IHM, LOHS, and costs were also calculated for each year under investigation. Costs were determined using diagnosis-related groups for the respective disease. Diagnosis-related groups are medical cost classifications that encompass a group of diseases managed using similar resources [23]. The hospitalizations were also categorized as urgent or planned.

Additionally, comorbidities were analyzed when EB was the primary diagnosis, as well as when it was the secondary diagnosis.

### Statistical Analysis

Quantitative variables are expressed as mean with standard deviation (SD) or median with interquartile range (IQR). Qualitative variables are expressed as frequencies and percentages or prevalence. Comparisons were performed using the χ2 test, Fisher’s exact test, t-test, or analysis of variance, as appropriate.

The total cost per inpatient was calculated. Given the skewed nature of cost data, the mean (SD), median, and 25th and 75th percentiles of the distribution were calculated. The costs per hospitalization were compared by sex, age group, CCI, and mortality.

## 3. Results

We identified a total of 677 discharges involving 347 EB patients from January 2016 to December 2021. In 2016, there were 94 hospital discharges (13.9%), followed by 86 (12.7%) in 2017, 132 (19.5%) in 2018, 119 (17.6%) in 2019, 141 (20.8%) in 2020, and 105 (15.5%) in 2021.

The full data set included thirty cases of EBS (8.6%), five of JEB (1.4%), ninety-two of DEB (26.5%), thirty-five of other EB (10.1%), and one hundred and eighty-five of nonspecific EB (53.3%). 

Table S3 shows the distribution of the EB subtypes according to the main study variables. As can be seen in this table, the subtypes with a code for “Other EB” were older than the other subtypes. The sex distribution was very similar for all subtypes with a similar prevalence in men and women. The proportion of patients that died during the hospital admission was also stable across the subtypes, with slightly higher values in those subtypes than the older patients.

Table 1 shows the distribution—according to the study variables and age group—of hospital discharges of patients with a diagnosis of EB in Spain from 2016 to 2021. 

Mean age was 24.9 years, and 50.7% were male. More than 50% (51.3%) experienced one readmission, while 31.2% had three or more readmissions. Rehospitalizations were more frequent among patients in the 2- to 17-year age group. 

The most frequent comorbidities were related to the digestive system (prevalence of 35%), infectious diseases (26.3%), and cancer (19.9%). The distribution of comorbidities with respect to age group is statistically significant. Gastrointestinal problems were most frequently observed in the 2- to 17-year age group. Over 40% of patients aged over 50 years were diagnosed with cancer.

The oldest age group had the highest burden of comorbidities. The CCI increased with age. No intrinsic CCI comorbidities were observed in infants, although the index increased with age, reaching 38.35% with a CCI >1 in the older age group (*p* < 0.005).

The mean global LOHS was 9.4 days, and the mean LOHS for admissions for EB differed significantly between the different age groups, with the shortest stay observed in the 18- to 50-year age group. Additionally, 30 patients died during the study period, and statistically significant differences were found in the crude IHM, which was higher in the older age group. However, this association must be interpreted with caution as it was not adjusted for confounding factors. As shown in Table 2, a total of 128 patients were diagnosed with at least one malignant neoplasm, resulting in a prevalence rate of 36.88%. Of these, seven patients were diagnosed with cancer on two separate occasions. At least one cutaneous squamous cell carcinoma (CSCC) was coded in 19.3% (67/347) of the study population, mostly affecting the lower limb, including the hip. The highest frequency of cancer was observed in patients within the age range of 18–50 years (73/128). More than 41% (53/128) of malignant neoplasms appeared in patients older than 50 years. Only three patients in the 2- to 17-year group developed cancer. Two were diagnosed with CSCC in the right upper limb, and the other had a malignant neoplasm in the cerebrum.

Table 3 shows the most common primary diagnoses of patients with EB in a secondary diagnostic position distributed by age range. The most frequent diagnosis was CSCC (8.3%), followed by “esophageal obstruction” (4.1%) and “certain localized infections” (3.4%). The table also shows the most common secondary diagnoses for patients discharged with a final diagnosis of EB. The most frequent diagnosis was “certain localized infections” (13.7%), “esophageal obstruction” (11.2%), “iron deficiency anemia and other common nutrition problems” (10%), and “dysphagia, oropharyngeal phase” (5.9%).

Table 4 summarizes the most frequent procedures performed on the study participants. The most common procedure was “excision or extraction of skin, external approach” (12.5%), followed by “transfusion, percutaneous approach” (8.4%), “dilation of esophagus” (6.2%), “replacement of skin with nonautologous/autologous tissue substitute, external approach” (5.2%), and “percutaneous endoscopic gastrostomy surgery” (3.9%).

Table 5 shows the cost per patient over the 6-year period according to several study variables. The overall mean cost per inpatient was estimated to be EUR 10,895.2 (SD EUR 13,394.8) in 2021. We found no statistically significant difference in cost by sex, although significant differences were observed in the cost of hospitalized patients by age group, with the 2- to 17-year age group being the most expensive in terms of hospitalization costs (EUR 19,601, SD EUR 18,675.7) compared to the 0- to 1-year age group, which generated the lowest expenditure (EUR 8923.1, SD EUR 14,013.8). Higher hospitalization costs were also recorded in patients who had an urgent hospitalization or died in the hospital compared to those with a planned admission or those who survived (*p* < 0.05). No correlation was observed between cost and the level of comorbidity (Table 5).

## 4. Discussion

Complications of EB are a major cause of hospitalization and rehospitalization [24]. There are currently no data on the rates of lifetime hospitalization of patients with underlying EB. Clinically stable patients with EB are usually treated in an interdisciplinary outpatient setting. Detection of CSCC, digestive tract problems, or whole-body infections are potentially life-threatening conditions that require urgent hospital admission and evaluation to prevent clinical deterioration. Therefore, the MBDS enables epidemiological analysis of severity in patients with EB, including factors such as rehospitalization and IHM, with a high degree of certainty.

Clinical research in EB faces important challenges, such as sample size and recruitment failures [25]. Among the participants included in the present study, the prevalence of hospitalizations was lowest in early childhood, increasing during adolescence and adulthood until the age of 50 years and decreasing thereafter. Whereas older patients with EB showed adverse in-hospital outcomes, such as death, patients in the 2- to 17-year age group were more likely to be readmitted. It is noteworthy that over 50% of the study population experienced their first readmission within a 6-year time frame. Subsequent rehospitalizations were also very frequent.

Comorbidity affects the prognosis of patients diagnosed with EB. In line with previous studies [26,27], the most frequent complications we recorded were conditions affecting the digestive tract, such as esophageal obstruction and anemia, and recurrent skin cancer. 

As patients become older, there is a progressive increase in comorbidities, as evidenced by higher scores on the CCI, which is due to the accumulation of chronic diseases, increased exposure to risk factors, increased frailty, and decreased physiological reserve [22]. 

According to our data, at least 36% of patients with EB developed cancer at some point in their lives (CSCC in approximately 50%). These percentages are lower than those published elsewhere [14], likely owing to sample size and long follow-up period. What is evident is the high lifetime risk of developing CSCC in patients with epidermolysis, suggesting that early detection of oncologic lesions is critical if we are to improve life expectancy and quality of life. In our study, the youngest patient with a malignant neoplasm was aged under 10 years. To date, we have not found any published cases of children with epidermolysis who developed comorbid cancer at such an early age. However, it is worth noting that the youngest patient diagnosed with CSCC was in the 2- to 17-year age group, consistent with the earliest reported instance of CSCC in a patient with basal cell cancer at the age of 12 years [28].

Of note, CSCC was the most frequent primary diagnosis associated with EB in our study. Chronic skin damage [28], heightened skin sensitivity, ongoing treatments, and recurrent wounds are significant risk factors contributing to the development of CSCC [15,29]. Given the higher risk of skin cancer in patients with EB, it is crucial for them to take additional precautions, such as avoiding excessive sun exposure, using broad-spectrum sunscreen, and regular consultation with dermatologists to detect and treat any suspicious lesions early [14].

It is worth noting that in our study, the most common secondary diagnosis associated with EB was skin infections. In EB, various microorganisms have been reported to colonize wounds, impede wound healing, and lead to serious bacterial infections [30].

As reported elsewhere [31,32], esophageal dilatation and percutaneous endoscopic gastrostomy were frequent procedures in our study population. EB can affect the mucosa of the entire body, including the lining of the gastrointestinal tract, leading to serious gastrointestinal complications that require the above procedures [26].

In a previous study [16], the direct healthcare costs of Spanish patients with EB were reported to be approximately EUR 4500 per patient per year. However, the current study revealed that the average total costs per hospitalized patient exceeded EUR 10,000. Angelis et al. focused on direct healthcare costs, which encompass outpatient visits, medications, and related expenses. Our study specifically examined the average total costs per inpatient, which incorporate a wider range of factors, such as hospital stays, surgeries, and intensive care. These disparities in cost figures can be attributed to differences in the methods used to calculate healthcare costs. These differences include variations in data collection methods, time frames considered, cost inclusion/exclusion criteria, patient characteristics, differences in patient populations (disease severity, specific subtypes of EB, and associated comorbidities). The differences may lead to variations in cost outcomes and in the healthcare system and resource utilization. It is crucial to take these factors into account when interpreting and comparing costs across studies. Furthermore, the variations highlight the complex nature of healthcare costs and the importance of taking a comprehensive approach to assessing the economic impact of EB on patients and healthcare systems. Our study acknowledges the skewed nature of cost data and presents descriptive statistics, although it does not provide a detailed analysis of cost drivers or potential confounding factors.

We were unable to compare our LOHS with that of previous publications on EB. Therefore, we refer to published data on rare diseases [33]. LOHS for rare diseases is reported as 6.7 days, with a longer duration observed in children aged under 5 years (8.7 days). Similarly, in our study population, we observed a longer average LOHS in children. However, the average LOHS in our EB population was significantly higher. 

Since the CCI has not been previously applied in the series of patients diagnosed with EB, it is impossible for us to make comparisons. Of note, the increase in the CCI with age does not necessarily imply a negative prognosis. The CCI is used as a risk stratification tool and is not an absolute predictor of mortality. Furthermore, proper medical care and management of chronic diseases can help improve health outcomes in these age groups. Assessment of comorbidity relies on diagnostic codes to identify those conditions associated with EB. However, the accuracy of identification of comorbidity based solely on diagnostic codes may be subject to coding errors or omissions. Additionally, the study focuses on specific comorbidities, and there may be other relevant comorbid conditions not included in the analysis.

Our study was conducted during the COVID-19 pandemic, a period characterized by important disruptions in the healthcare system at the beginning of 2020 in many countries, including Spain. While our research did not specifically address the pandemic’s impact, it’s possible that some observed results were influenced by these exceptional circumstances. In fact, the greatest number of hospital admissions of patients with EB was found for the year 2020 (141). Therefore, the COVID-19 pandemic probably had a major effect on the data from the year 2020. A follow-up analysis in the upcoming years is required to estimate the long-term effect of the COVID-19 pandemic on EB.

In addition to the shortcomings discussed above, our study is subject to other, important limitations. The first is diagnostic accuracy, where misdiagnosis or underdiagnoses of cases of EB may affect the reliability and accuracy of the epidemiological analysis. Furthermore, we have a very significant proportion of cases with unspecified subtypes (53%) or codified as “other EB” (10%), and within some specific subtypes, there are very few patients (thirty cases of EBS and five of JEB). This high proportion of unspecified subtypes and the small sample size of some subtypes limit the statistical validity of a stratified analysis and make it difficult to obtain reliable results. A second limitation is the sample size, which was small owing to the relative rarity of EB and the limited number of cases available for analysis, thus restricting the generalizability and statistical power of the results. The third limitation is the data source; the study uses data from the MBDS, which incorporates data from public and private hospitals and covers a high percentage of hospital discharges. However, it is important to remember that the database may not capture all of the cases of EB, especially those that do not result in hospitalization or are treated in outpatient settings. This could introduce selection bias and limit the representativeness of the findings. Finally, in our study, we do not aim to provide incidence rates or to conduct a time-dependent analysis as we do not have information on the moment of the first diagnosis or the first hospitalization of patients with EB, since these two events may have occurred before the year 2016. We simply aim to describe the number of hospitalizations with EB in Spain over a 6-year period and to identify the total number of affected patients.

Nevertheless, our research is pioneering in that it is the first of its kind to investigate epidemiological trends and hospital outcomes related to EB at the national level.

Overall, our study emphasizes the importance of accurate epidemiological data when investigating rare diseases such as EB, identifying associated comorbidities, and optimizing medical care. It also underscores the need for early detection of complications, such as CSCC and other malignant neoplasms, and the implementation of preventive measures to improve the quality of life and prognosis of EB patients.

## 5. Conclusions

In conclusion, to our knowledge, this is the first study to provide valuable insights into epidemiological patterns and hospital outcomes related to EB in Spain. Readmissions are very frequent among people with EB. We observed a higher LOHS in the 0- to 1-year age group, while the highest rates of IHM were observed in patients older than 50 years. Additionally, it is remarkable to observe the high prevalence of comorbidities in EB, the most common of which are digestive disorders, infectious diseases, and cancer. Furthermore, this study delves into the economic implications of hospitalizations for EB, revealing significant costs associated with inpatient care. Our findings highlight the burden of EB on patients and healthcare systems, emphasizing the need for specialized medical care and comprehensive support for patients affected by this rare disease.

## Figures and Tables

**Table 1:** Distribution according to study variables and age groups of hospital discharges with a diagnosis of epidermolysis bullosa in Spain from 2016 to 2021.

Age Group	0–1	2–17	18–50	>50	Total
Number of patients, *n* (%)	89 (25.6)	49 (14.1)	116 (33.4)	93 (26.8)	347 (100)
Number of hospitalizations. Total, *n* (%) *	120 (17.7)	204 (30.1)	220 (32.5)	133 (19.6)	677 (100)
Men, *n* (%) Women, *n* (%)	64 (53.3)	78 (38.2)	132 (60)	69 (51.9)	343 (50.6)
	56 (46.7)	126 (61.8)	88 (40)	64 (48.1)	334 (49.3)
Number of Hospital admissions One, *n* (%) Two, *n* (%) Three or more, *n* (%)					
	89 (74.2)	49 (24)	116 (52.7)	93 (69.9)	347 (51.3)
	16 (13.3)	47 (23)	37 (16.8)	19 (14.3)	119 (17.5)
	15 (12.5)	108 (52.9)	67 (30.5)	21 (15.8)	211 (31.2)
Age, mean (SD) *	0.2 (0.9)	9.3 (4.2)	27.1 (11.8)	67.9 (13)	24.9 (25.11)
Acute Myocardial Infarction, *n* (%)	0 (0)	0 (0)	1 (0.5)	4 (3)	5 (0.7)
Congestive Heart, *n* (%) *	0 (0)	17 (8.3)	5 (2.3)	20 (15)	42 (6.2)
Peripheral Vascular diseases, *n* (%) *	0 (0)	0 (0)	0 (0)	9 (6.8)	9 (1.3)
Cerebrovascular diseases, *n* (%) *	0 (0)	0 (0)	2 (0.9)	12 (9)	14 (2.1)
Dementia, *n* (%) *	0 (0)	0 (0)	0 (0)	7 (5.3)	7 (1)
Chronic Obstructive Pulmonary, *n* (%) *	0 (0)	0 (0)	10 (4.6)	13 (9.8)	23 (3.4)
Rheumatoid Disease, *n* (%)	0 (0)	0 (0)	1 (0.5)	4 (3)	5 (0.7)
Peptic Ulcer, *n* (%)	0 (0)	0 (0)	0 (0)	1 (0.8)	1 (0.2)
Liver disease, *n* (%) *	0 (0)	0 (0)	10 (4.5)	24 (18)	34 (5)
Diabetes, *n* (%) *	0 (0)	0 (0)	3 (1.4)	28 (21.1)	31 (4.6)
Hemiplegia or Paraplegia, *n* (%)	0 (0)	0 (0)	2 (0.9)	4 (3)	6 (0.9)
Renal diseases, *n* (%) *	0 (0)	8 (3.9)	14 (6.4)	27 (20.3)	49 (7.2)
Cancer, *n* (%) *	0 (0)	3 (1.5)	74 (33.6)	58 (40.6)	135 (19.9)
AIDS, *n* (%)	0 (0)	0 (0)	5 (2.3)	1 (0.8)	6 (0.9)
Hypertension, *n* (%) *	0 (0)	8 (0)	16 (7.3)	59 (44.4)	83 (12.3)
Alcohol Abuse, *n* (%)	0 (0)	0 (0)	4 (1.8)	7 (5.3)	11 (1.6)
Digestive system diseases, *n* (%) *	15 (12.5)	119 (58.3)	61 (27.7)	42 (31.6)	237 (35)
Respiratory system diseases, *n* (%) *	18 (15)	12 (5.9)	23 (10.5)	31 (23.3)	84 (12.4)
Infectious diseases, *n* (%) *	35 (29.2)	48 (23.5)	45 (20.5)	50 (37.6)	178 (26.3)
External causes, *n* (%) *	4 (3.3)	27 (13.2)	24 (10.9)	32 (24.1)	87 (12.9)
Musculoskeletal system diseases *	1 (0.8)	12 (5.9)	14 (6.4)	34 (25.6)	61 (9)
Charlson Comorbidity Index None, *n* (%) * One, *n* (%) * Two or more *n* (%) *					
	120 (100)	185 (90.7)	179 (81.4)	47 (35.3)	530 (78.3)
	0 (0)	11 (5.4)	26 (11.8)	35 (26.3)	73 (10.8)
	0 (0)	8 (3.9)	15 (6.8)	51 (38.4)	74 (10.9)
LOHS mean (SD) (median [IQR]) *	14.5 (17.7) (8 [15.5])	8.1 (15.2) (3 [6])	6.9 (13.7) (3 [5])	10.9 (18.3) (6 [9])	9.4 (16.1) (4 [8])
In-hospital mortality, *n* (%) *	6 (5)	2 (1)	8 (3.6)	14 (10.5)	30 (4.4)

* *p* < 0.05: statistically significant association comparing the different age groups. LOSH: length of hospital stay.

**Table 2:** Frequency of types of cancer in patients in the study population.

Malignant Neoplasm Types		*n*	Prevalence (%)
Skin cancer	Squamous cell carcinoma (SCC)	67	19.3
	Basal cell carcinoma (BCC)	1	0.3
	Secondary malignant neoplasm of skin	1	0.3
Breast cancer		4	1.2
Prostate cancer		2	0.5
Hematologic malignancies		3	0.9
Colorectal cancer		7	2
Secondary liver cancer		7	2
Secondary endocrine cancer		5	1.4
Secondary bone cancer		3	0.9
Secondary lung cancer		7	2
Secondary lymphatic system cancer		7	2
Others		14	4
Total		128	36.88

**Table 3:** The most frequent primary and secondary diagnosis in patients with final diagnosis of inherited epidermolysis bullosa.

	Age Group (Years)				
PRIMARY DIAGNOSIS	0–1	2–17	18–50	>50	Total, *n* (%)
Esophageal obstruction, *n*	0	25	12	2	28 (4.1)
Squamous cell carcinoma of skin, *n*	0	2	48	7	56 (8.3)
Other Malignant neoplasms, *n*	0	1	2	7	10 (1.4)
Certain localized infections, *n*	3	7	5	8	23 (3.4)
Pneumonia, unspecified organism	0	0	5	3	8 (1.2)
SECONDARY DIAGNOSIS					
Iron deficiency anemia and other common nutrition problems, *n*	4	24	27	13	68 (10)
Dysphagia, oropharyngeal phase, *n*	4	12	18	6	40 (5.9)
Certain localized infections, *n*	21	26	24	22	93 (13.7)
Ascites, *n*	0	0	1	10	11 (1.6)
Severe sepsis, *n*	0	6	3	6	15 (2.29)

**Table 4:** The most frequent procedures in hospitalizations of patients with a final diagnosis of inherited epidermolysis bullosa.

Procedures	*n*-Hospitalizations (%)
Dilation of Esophagus	62 (6.2)
Percutaneous Endoscopic Gastrostomy Surgery	39 (3.9)
Excision or Extraction of Skin, External Approach	125 (12.5)
Replacement of Skin with Nonautologous/Autologous Tissue Substitute, External Approach	52 (5.2)
Extraction and Delivery of Products of Conception	15 (1.5)
Transfusion, Percutaneous Approach	84 (8.4)

**Table 5:** Total hospitalization cost during the 6-year study period.

Variable	Categories	Inpatient Hospitalization Cost Mean (SD) (Median [25th and 75th Percentiles]), €
Sex	Men	11,595.2 (14,224) (5825.3 [3038.8–15,374.5])
	Women	10,095.9 (12,375.9) (5393.9 [3038.8–10,938.2]
Age groups (years)	0–1	8923.1 (14,013.8) (3038.8 [1922.6–9417.6])
	2–17	19,601.8 (18,675.7) (10,272.8 [7252.3–26,184.5])
	18–50	10,336.3 (11,942.5) (5009.7 [3001–14,614.3])
	50	9143.31 (9552.55) (5414.95 [3726.33–10,751.78])
Charlson Comorbidity index	0	10,352.89 (12,852.59) (5363.26 [2662.06–11,637.54])
	1	10,371.48 (9008.83) (6635.49 [4270.57–14,669.65])
	2 or more	15,033.9 (19,692.52) (6642.52 [4079.20–17,877.58])
Hospital admission	Urgent	12,799.1 (12,611.4) (6706 [3387.2–14,649.2]
	Planned	8312.9 (11,221.7) (5809 [2519.1–12,860.1]
In-hospital mortality	Yes	19,144.2 (19,103.4) (14,169.5 [5543.1–25,471.6])
	No	10,254.8(12,664.9) (5363.3 [3013.9–10,929.5])
Total cost per patient		10,895.2 (13,394.8) (5522.7 [3038.8–12,665.7])

## Data Availability

According to the contract signed with the Spanish Ministry of Health and Social Services, which provided access to the databases from the minimum basic data set (MBDS), we cannot share the databases with any other investigator, and we have to destroy the databases once the investigation has concluded. Consequently, we cannot upload the databases to any public repository. However, any investigator can apply for access to the databases by filling out the questionnaire available at https://www.sanidad.gob.es/estadEstudios/estadisticas/estadisticas/estMinisterio/SolicitudCMBD.htm (accessed on 20 May 2023). All other relevant data are included in the paper.

## Supplementary Materials

The following supporting information can be downloaded at: https://www.mdpi.com/article/10.3390/biomedicines11092584/s1. Table S1: Specific comorbidities and malignant neoplasm types identified in this investigation with their corresponding ICD-10-CM codes. Table S2: Procedures conducted during the hospitalization are identified in this investigation with their corresponding ICD-10-CM codes. Table S3: Distribution of epidermolysis bullosa (EB) subtypes according to the main study variables.
