# Supplementary material for: Clinical Characteristics and Outcomes of Patients Hospitalized with Epidermolysis Bullosa: A Retrospective Population-Based Observational Study in Spain (2016–2021)
Source: Biomedicines. 2023 Sep 20;11(9):2584. doi: 10.3390/biomedicines11092584 (PMC10526251; doi:10.3390/biomedicines11092584)
Supplement: Supplementary file 1 [file biomedicines-11-02584-s001.zip › Table S1. Biomedicine.pdf]

Supplementary Table S1. Specific comorbidities and malignant neoplasm types identified in this investigation with their corresponding ICD-10-CM codes.

DIAGNOSIS	ICD-10-CM Codes
Digestive system	K00-K95, Q39.3, 8, Q40.0, Q61.2, Z43.1, Z 43.2
Respiratory system	J00-J99
Infectious and parasitic	A00-B99, G04.90, O23.01, L00-L08
Injury, poisoning and certain other consequences of external causes	S00 -T88
Musculoskeletal system	M00-M99
Squamous cell carcinoma	C44.329, C44.42, C44.529, C44.622, C44.629, C44.721, C44.722, C44.729, D04.62
Basal cell carcinoma	C44.91
Secondary malignant neoplasm of skin	C79.2
Breast cancer	C50.011, C50.912, D05.11
Prostate cancer	C61
Hematologic malignancies	C91.02, C92.10, D45
Colorectal cancer	C18.7, C20
Secondary liver cancer	C78.6, C78.7
Secondary endocrine cancer	C79.70, C79.71, C79.72, C7A.8
Secondary bone cancer	C79.51
Secondary lung cancer	C78.00, C78.01, C78.02
Secondary lymphatic system cancer	C77.1, C77.2, C77.3, C77.4, C77.9
Remaining cancers not included in the previous ones	C55, C71.0, C79.31, C90.00, D46.A, D47.2, C46.0, C79.89, C80.1
