# Supplementary material for: Clinical Characteristics and Outcomes of Patients Hospitalized with Epidermolysis Bullosa: A Retrospective Population-Based Observational Study in Spain (2016–2021)
Source: Biomedicines. 2023 Sep 20;11(9):2584. doi: 10.3390/biomedicines11092584 (PMC10526251; doi:10.3390/biomedicines11092584)
Supplement: Supplementary file 1 [file biomedicines-11-02584-s001.zip › Table S2. Biomedicine.pdf]

Supplementary Table S2. Procedures conducted during the hospitalization identified in this investigation with their corresponding ICD-10-CM codes.

Procedures	ICD-code 10
Dilation of Esophagus	0D718ZZ, 0D728ZZ, 0D738DZ, 0D757ZZ, 0D758DZ, 0D758ZZ
Percutaneous Endoscopic Gastrostomy Surgery	0DH60UZ, 0DH63UZ, 0DH64UZ, 0DH67UZ, 0DH673Z, 0DH68UZ, 0DHA7UZ, 0DHA3UZ
Excision or Extraction of Skin, External Approach	0HB0XZZ, 0HB1XZZ, 0HB4XZX, 0HB5XZX, 0HB6XZZ, 0HB7XZX, 0HB8XZX, 0HB8XZZ, 0HBBXZZ, 0HBCXZX, 0HBDXZX, 0HBFXZX,0HBFXZZ, 0HBGXZZ, 0HBHXZZ, 0HBJXZX, 0HBKXZX, 0HBKXZZ, 0HBLXZX, 0HBLXZZ, 0HBMXZX, 0HBMXZZ, 0HBNXZZ, 0HCLXZZ, 0HDFXZZ, 0HDGXZZ, 0HDNXZZ, 0HJPXZZ, 0HNFXZZ, 0HNGXZZ, 0HQFXZZ
Replacement of Skin with Nonautologous/ Autologous Tissue Substitute, External Approach	0HR6XK4, 0HRFX73, 0HRFX74, 0HRGX73, 0HRGX74, 0HRKXJ4, 0HRLX73, 0HRLX74, 0HRMX74, 0HRMXJZ, 0HRNX74, 0HRNX74, 0HR5XJ4, 0HRCX74, 0HREX74, 0HRKX73, 0HRKX74, 0HRLX74, 0HR5X74, 0HR7XJ4, 0HRFXJZ, 0HRFXK3, 0HRKX74, 0HRBXJ4, 0HRGXJ4, 0HRLX74, 0HRGXJZ, 0HRHXJ4, 0HRMXK3
Extraction and Delivery of Products of Conception	10D00Z1, 10D07Z6, 10D17ZZ, 10E0XZZ
Transfusion, Percutaneous Approach	30233N1, 30233R1, 30233Y2, 30243N1, 30243Y2, 30233K1, 30233P1, 30233R1, 30233S1, 30243N1, 30243J1, 30243R1, 30233L1
Skin care treatments and procedures	F08F5BZ, F08G5BZ, F08G5ZZ, F08H5BZ, F08H5ZZ, F08M5YZ, F08F5ZZ, F08H5FZ, F08K5ZZ, F08D5BZ, F08K5CZ, F08M5BZ
