# Supplementary material for: Clinical Characteristics and Outcomes of Patients Hospitalized with Epidermolysis Bullosa: A Retrospective Population-Based Observational Study in Spain (2016–2021)
Source: Biomedicines. 2023 Sep 20;11(9):2584. doi: 10.3390/biomedicines11092584 (PMC10526251; doi:10.3390/biomedicines11092584)
Supplement: Supplementary file 1 [file biomedicines-11-02584-s001.zip › Table S3. Biomedicine.pdf]

Supplementary Table S3. Distribution of epidermolysis bullosa (EB) subtypes according to main study variables

Epidermolysis bullosa subtypes		EBS	JEB	DEB	Other EB	Nonspecific EB
Individuals patients, No. (%)		30 (8.6)	5 (1.4)	92 (26.5)	35 (10.1)	185 (53.3)
Age groups, No. (%)	0-1 years	13 (43.3)	4 (80)	16 (17.4)	3 (8.6)	49 (26.5)
	2-17 years	3 (10)	0 (0)	41 (44.6)	1 (2.9)	40 (21.6)
	18-50 years	3 (10)	0 (0)	32 (34.8)	12 (34.3)	43 (23.2)
	>50 years	11 (36.7)	1 (20)	3 (3.3)	19 (54.3)	53 (28.6)
Sex, No. (%)	Men	14 (46.6)	2 (40)	53 (57.6)	20 (57.1)	98 (53)
	Women	16 (53.3)	3 (60)	39 (42.4)	15 (42.9)	87 (47)
In hospital mortality, No. (%)		3 (10)	1 (20)	7 (7.6)	5(14.3)	14 (7.6)

EBS epidermolysis bullosa simplex. JEB junctional epidermolysis bullosa. DEB dystrophic epidermolysis bullosa
